# Cigarette smoking behaviors and the importance of ethnicity and genetic ancestry

**DOI:** 10.1038/s41398-021-01244-7

**Published:** 2021-02-11

**Authors:** Hélène Choquet, Jie Yin, Eric Jorgenson

**Affiliations:** Division of Research, Kaiser Permanente Northern California (KPNC), Oakland, CA 94612 USA

**Keywords:** Pathogenesis, Genetics

## Abstract

Cigarette smoking contributes to numerous diseases and is one of the leading causes of death in the United States. Smoking behaviors vary widely across race/ethnicity, but it is not clear why. Here, we examine the contribution of genetic ancestry to variation in two smoking-related traits in 43,485 individuals from four race/ethnicity groups (non-Hispanic white, Hispanic/Latino, East Asian, and African American) from a single U.S. healthcare plan. Smoking prevalence was the lowest among East Asians (22.7%) and the highest among non-Hispanic whites (38.5%). We observed significant associations between genetic ancestry and smoking-related traits. Within East Asians, we observed higher smoking prevalence with greater European (versus Asian) ancestry (*P* = 9.95 × 10^−12^). Within Hispanic/Latinos, higher cigarettes per day (CPD) was associated with greater European ancestry (*P* = 3.34 × 10^−25^). Within non-Hispanic whites, the lowest number of CPD was observed for individuals of southeastern European ancestry (*P* = 9.06 × 10^−5^). These associations remained after considering known smoking-associated loci, education, socioeconomic factors, and marital status. Our findings support the role of genetic ancestry and socioeconomic factors in cigarette smoking behaviors in non-Hispanic whites, Hispanic/Latinos, and East Asians.

## Introduction

Cigarette smoking contributes to numerous common diseases, including cancers, chronic obstructive pulmonary disease, and cardiovascular diseases, and it is one of the leading causes of death in the United States^1–6^. Despite the substantial decrease in cigarette smoking prevalence over the last one-half century, ~40 million people are still smokers in the United States, and disparities among smokers remain^7,8^. Higher prevalences of smokers have been observed in populations who are disadvantaged socially and economically^7,9^. Further, among smokers, socioeconomic status is a major determinant of the degree of nicotine dependence^10^, which can be approximated by the number of cigarettes smoked per day (CPD)^11^.

In the United States, smoking behaviors vary widely across race/ethnicity, with individuals of Asian and Hispanic/Latino ancestry having the lowest smoking prevalence compared to individuals of other ancestry^7,8^. The reasons for these disparities may include variation in genetic ancestry, which has the potential to explain variation in smoking behaviors between Asian and Hispanic/Latino ancestry populations and other populations. However, to date, no study has investigated the role of genetic ancestry and smoking behavior-related traits.

Twin and family studies suggest that genetic factors accounted for approximately half of the variance in smoking initiation and smoking quantity, and heritable variation in cigarette use seems comparable across ethnic groups^12–14^. Recently, the GWAS and Sequencing Consortium of Alcohol and Nicotine Use (GSCAN) study^15^ conducted in European ancestry individuals reported 467 genetic variants associated with cigarette smoking-related traits, including age at smoking initiation, smoking initiation, smoking cessation, and CPD.

Here, we hypothesize that genetic ancestry may explain some of the wide-variability in cigarette smoking behaviors across ethnic groups. To answer this question, we conduct genetic ancestry analyses of cigarette smoking behaviors within each of the four ethnic groups (non-Hispanic whites, Hispanic/Latinos, East Asians, and African Americans) from the Genetic Epidemiology Research in Adult Health and Aging (GERA) cohort^16^. Two smoking-related traits were used: smoking initiation (15,862 ‘ever’ smokers vs. 27 623 ‘never’ smokers) and CPD for all smokers (i.e., 2271 ‘current’ + 13,591 ‘formers’ smokers). We then investigate whether genetic ancestry associations are: (1) due to genetically determined smoking-related traits based on known smoking genetic variants^15^; and (2) modified by education, socioeconomic factors such as, employment/work status, household income, and marital status.

## Materials and methods

### Study population

Individuals were selected from the Kaiser Permanente Research Program on Genes, Environment, and Health (RPGEH) Genetic Epidemiology Research on Adult Health and Aging (GERA) cohort. The cohort consists of over 110,000 adult members of Kaiser Permanente Northern California (KPNC), ranging in age from 18 to 100 years at enrollment^16^. The RPGEH was established as a resource for research on genetic and environmental influences on health and disease, and participants were asked to complete a mailed survey. On this survey, participants were asked: ‘What best describes your race/ethnicity?’. Briefly, and as previously described^16^, self-reported race/ethnicity for each individual was derived from responses to this question, and, for individuals who reported more than one category, the selections were collapsed into race/ethnicity categories. In particular, all East Asian nationalities (i.e., Chinese, Japanese, Korean, Filipino, Vietnamese, or other Southeast Asian) were collapsed into a single East Asian group; all Latino nationalities (i.e., Mexican, Central/South American, Puerto Rican, or other Latino/Hispanic) were collapsed into a single Hispanic/Latino category; all African descent populations (i.e., African-American, African, or Africo-Caribbean) were collapsed into a single group; all white-European ethnicities (i.e., White or European-American, Middle Eastern, or Ashkenazi Jewish) were collapsed into a single non-Hispanic white group. In addition to self-reported race/ethnicity, individuals included in the current study provided self-reported information regarding their cigarette use, education, employment/work status, household income, and marital status (*N* = 43,485, Table 1). All study procedures were approved by the Institutional Review Board of the Kaiser Foundation Research Institute.

**Table 1:** Characteristics of the GERA participants included in the current study.

	All	NHW	H/L	EAS	AA	*P*-value
*N* (%)	43,485 (100%)	33,538 (77.1)	4392 (10.1)	4052 (9.3)	1503 (3.5)	–
Age (years) mean ± sd	54.8 ± 11.3	55.7 ± 10.9	51.4 ± 12.5	51.6 ± 11.9	53.9 ± 11.6	1.06 × 10^−203^
Female *n* (%)	25,140 (57.8)	19,312 (57.6)	2621 (59.7)	2319 (57.2)	888 (59.1)	0.0361
Never smokers *n* (%)	27,623 (63.5)	20,640 (61.5)	2903 (66.1)	3131 (77.3)	949 (63.1)	2.27 × 10^−86^
Ever smokers *n* (%)	15,862 (36.5)	12,898 (38.5)	1489 (33.9)	921 (22.7)	554 (36.9)	
CPD mean ± sd	20.4 ± 9.4	21.2 ± 9.4	17.0 ± 8.6	16.4 ± 8.4	17.1 ± 8.0	7.30 × 10^−110^
Former smokers *n* (%)	13,591 (31.3)	11,181 (33.3)	1209 (27.5)	779 (19.2)	422 (28.1)	1.48 × 10^−16^
CPD mean ± sd	20.7 ± 9.5	21.5 ± 9.5	17.3 ± 8.7	16.7 ± 8.6	17.6 ± 8.2	1.50 × 10^−88^
Current smokers *n* (%)	2271 (5.2)	1717 (5.2)	280 (6.4)	142 (3.5)	132 (8.7)	–
CPD mean ± sd	18.3 ± 8.3	19.2 ± 8.4	15.7 ± 7.8	14.9 ± 7.2	15.4 ± 7.3	3.28 × 10^−17^
Education *n* (%)						3.00 × 10^−286^
Less than high school	190 (0.4)	39 (0.1)	105 (2.4)	38 (0.9)	8 (0.5)	
High school	3222 (7.4)	2177 (6.5)	679 (15.5)	252 (6.2)	114 (7.6)	
Some college	9858 (22.7)	7534 (22.5)	1252 (28.5)	609 (15.0)	463 (30.8)	
College degree or more	30,215 (69.5)	23,788 (70.9)	2356 (53.6)	3153 (77.8)	918 (61.1)	
Employment *n* (%)						6.39 × 10^−43^
Full-time employed	33,231 (76.4)	25,239 (75.3)	3439 (78.3)	3312 (81.7)	1241 (82.6)	
Part-time employed	8099 (18.6)	6631 (19.8)	716 (16.3)	588 (14.5)	164 (10.9)	
Disabled	1234 (2.8)	987 (2.9)	142 (3.2)	41 (1.0)	64 (4.3)	
Unemployed	921 (2.1)	681 (2.0)	95 (2.2)	111 (2.7)	34 (2.3)	
Income per year level *n* (%)						6.98 × 10^−96^
<$20,000	1061 (2.4)	713 (2.1)	188 (4.3)	107 (2.6)	53 (3.5)	
$20,000–$59,999	8433 (19.4)	5993 (17.9)	1217 (27.7)	777 (19.2)	446 (29.7)	
$60,000+	33,991 (78.2)	26,832 (80.0)	2987 (68.0)	3168 (78.2)	1004 (66.8)	
Marital status *n* (%)						1.34 × 10^−96^
Never married	5241 (12.1)	3617 (10.8)	706 (16.1)	638 (15.7)	280 (18.6)	
Married or living as married	31,774 (73.1)	24,824 (74.0)	3049 (69.4)	3047 (75.2)	854 (56.8)	
Separated/divorced	6470 (14.9)	5097 (15.2)	637 (14.5)	367 (9.1)	369 (24.6)	

*NHW* non-Hispanic whites, *H/L* Hispanic/Latinos, *EAS* East Asians, *AA* African Americans, *CPD* number of cigarettes smoked per day.

### Smoking-related traits

Two smoking-related traits (i.e., smoking initiation, and the number of CPD) were assessed based on the RPGEH survey, via the following questions: ‘Have you ever smoked one or more cigarettes per day for six months or longer?’ (yes or no); ‘Do you currently smoke, or have you stopped smoking?’ (current smoker or former smoker); and ‘On average how many packs of cigarettes do you (or did you) smoke per day?’(< ½ pack, ½–1 pack, 1–1½ packs, or more than 1½ packs). For smoking initiation, ever (former/current) and never smokers were assigned as cases and controls, respectively. For smokers (‘former’ and “current’ smokers), the number of CPD, as a quantitative trait, was assessed by considering ~20 cigarettes per pack. The RPGEH survey has been shown to be successful in assessing other substance use, such as alcohol consumption, as in our recent study^17^ we confirmed previous findings implicating *ADH1B*, *AUTS2*, *SGOL1*, *SERPINC1*, *KLB*, and *GCKR* loci in alcohol consumption^18–21^.

### Socioeconomic covariates

The RPGEH survey was also used to assess education, socioeconomic factors (i.e., employment/work status and household income), and marital status, via the following questions: ‘What is the highest level of school that you have completed?’; ‘What is your employment or work status?’; ‘What best describes your household income (before taxes)?’; and ‘What is your current marital status?’. Answers to these questions were combined in: (1) 4 categories for education: ‘less than high school’ which corresponds to “grade school (grades 1–8)”, ‘high school’ which combines “some high school (grades 9–11)” with “high school or GED”, ‘some college’, and ‘college degree or more’ which combines “college”, “graduate school”, and “technical/trade school”; (2) 4 categories for employment or work status: ‘full-time employed’, ‘part-time employed’, ‘unemployed’ and ‘disabled for work’; (3) 3 categories for household income: ‘<$20,000’ which corresponds to an annual household income (before taxes) <$19,999 per year, ‘$20,000 to $59,999/year’, and ‘$60,000/year or more’; and (4) 3 categories for marital status: ‘never married’, ‘married or living as married’, and ‘separated or divorced’. ‘Female’ sex, ‘college or more’ education, ‘$60,000 or more’ income, ‘full-time employed’ employment, and ‘married or living as married’ marital status served as the reference groups for Models 3.

### Genotyping and imputation

GERA DNA samples were genotyped on four custom Affymetrix Axiom arrays that were designed for individuals of non-Hispanic white, East Asian, African American, and Latino race/ethnicity, as previously described^22,23^. We applied genotype quality control (QC) procedures for the GERA samples on an array-wise basis^23^. Briefly, we included genetic markers with an initial genotyping call rate ≥97%, genotype concordance rate >0.75 across duplicate samples, and allele frequency difference ≤0.15 between females and males for autosomal markers.

Approximately 94% of samples and more than 98% of genetic markers assayed reached QC procedures. In total, over 665,000 genotyped single nucleotide polymorphisms (SNPs)^22,24^ and over 15,000,000 imputed SNPs were available for analyses. The 1000 Genomes reference panel (phase I integrated release, March 2012) was used for imputation (IMPUTE2 v2.3.0, SHAPEIT v2.r72719).

### Principal component (PC) and genetic ancestry

Banda et al.^16^ conducted an analysis of ancestry in GERA using PC analysis (Eigenstrat v4.2), and identified 10 and 6 ancestry PCs reflecting genetic ancestry among non-Hispanic whites, and the other ethnic groups, respectively. To adjust for genetic ancestry, we also included the percentage of Ashkenazi (ASHK) Jewish ancestry as a covariate for the non-Hispanic white ethnic group analysis. For genetic ancestry analyses, for each ethnic group, we examined the effect of the first 2 PCs, which are the only ones geographically interpretable and represent geographic clines, on smoking-related traits prevalence/distribution. Each model was adjusted for additional PCs (i.e., up to 10 for non-Hispanic whites and up to 6 for the other ethnic groups). To visualize the smoking-related traits prevalence/distribution by the ancestry PCs, we created a smoothed distribution of each individual’s smoking phenotype using a radial kernel density estimate, as previously described^25^.

### Genetic risk score (GRS)

To determine if known smoking-associated SNPs could explain the ancestry effect, we repeated the ancestry analyses including a GRS for each smoking-related trait based on the findings of the largest genetic study conducted to date, including up to 1.2 million individuals with information on multiple stages of tobacco use^15^. To derive the GRS, we used a ‘classic’ method^26^ which consists of computing GRS based on a subset of SNPs exceeding a specific GWAS association *P*-value threshold (i.e., *P* ≤ 5.0 × 10^−8^ in Liu et al.^15^). The first GRS was based on 365 smoking initiation genome-wide associated-SNPs associated-SNPs, and the second was based on 53 SNPs previously reported to be associated at a genome-wide level of significance with CPD^15^. Out of the 365 SNPs, 133 (36.4%) were confirmed to be associated with smoking initiation in GERA, including 14 at a Bonferroni-corrected alpha level of 1.37 × 10^−4^ (0.05/365) (Supplementary Data 1). Out of the 53 SNPs, 34 (64.1%) were confirmed to be associated with CPD in GERA, including 15 at a Bonferroni-corrected alpha level of 9.43 × 10^−4^ (0.05/53) (Supplementary Data 2). The GRSs were built on these known smoking-associated SNPs by summing up the additive coding of each SNP weighted by the effect size ascertained from the original study^15^. As the original study^15^ was conducted in cohorts of European ancestry, we also generated unweighted GRSs and included those in the models for each ethnic group. Results were similar using unweighted or weighted GRS in all ethnic groups (Supplementary Data 3).

### Statistical analyses

For smoking initiation, we used a logistic regression model to examine the impact of ancestry on this smoking-related trait using R version 3.4.1 with the following covariates: age, sex, and ancestry PCs (first 10 PCs for the non-Hispanic white analyses and first 6 PCs for the other ethnic groups) (Model 1). For the number of CPD, we used a linear regression model. In Model 2, in addition to all covariates included in Model 1, we added one of the two GRS described above. In Model 3, in addition to all covariates included in Model 2, we added education, socioeconomic factors, and marital status as covariates.

## Results

### GERA cohort and smoking behavior

The study sample consisted of 43,485 GERA participants from four ethnic groups (non-Hispanic whites, Hispanic/Latinos, East Asians, and African Americans) (Table 1). In our study, the prevalence of ‘ever’ smokers varied by ethnicity with the lowest prevalence (22.7%) for East Asians and the highest (38.5%) for non-Hispanic whites. On average, the number of cigarettes per day (CPD) smoked by non-Hispanic whites was higher (21.2 CPD) compared to the number of CPD smoked by individuals from other ethnic groups (range of 16.4–17.1 CPD). ‘Ever’ smokers were more likely to be ‘former’ smokers compared to ‘current’ smokers in all ethnic groups.

In our study, the prevalence of ‘ever’ smokers also varied by education level, employment, income level, and marital status (Supplementary Table 1). Individuals with high school education levels were more likely to have smoked compared to individuals with a college degree or higher education level (51.3% vs. 31.7%). Individuals who were disabled were more likely to have smoked compared to individuals who were part- or full-time employed (53.3% vs. (34.8–36.1%)), and individuals having an annual income of $60,000 or more were less likely to have smoked compared to individuals who had an annual income of <$59,999 (34.5 vs. 43.6%). Finally, individuals who were separated/divorced were more likely to ever smoked compared to individuals who were never married (45.7% vs. 28.9%). Similar trends were observed across the four ethnic groups (Supplementary Table 2).

### Genetic ancestry and smoking behaviors

**Fig. 1:**
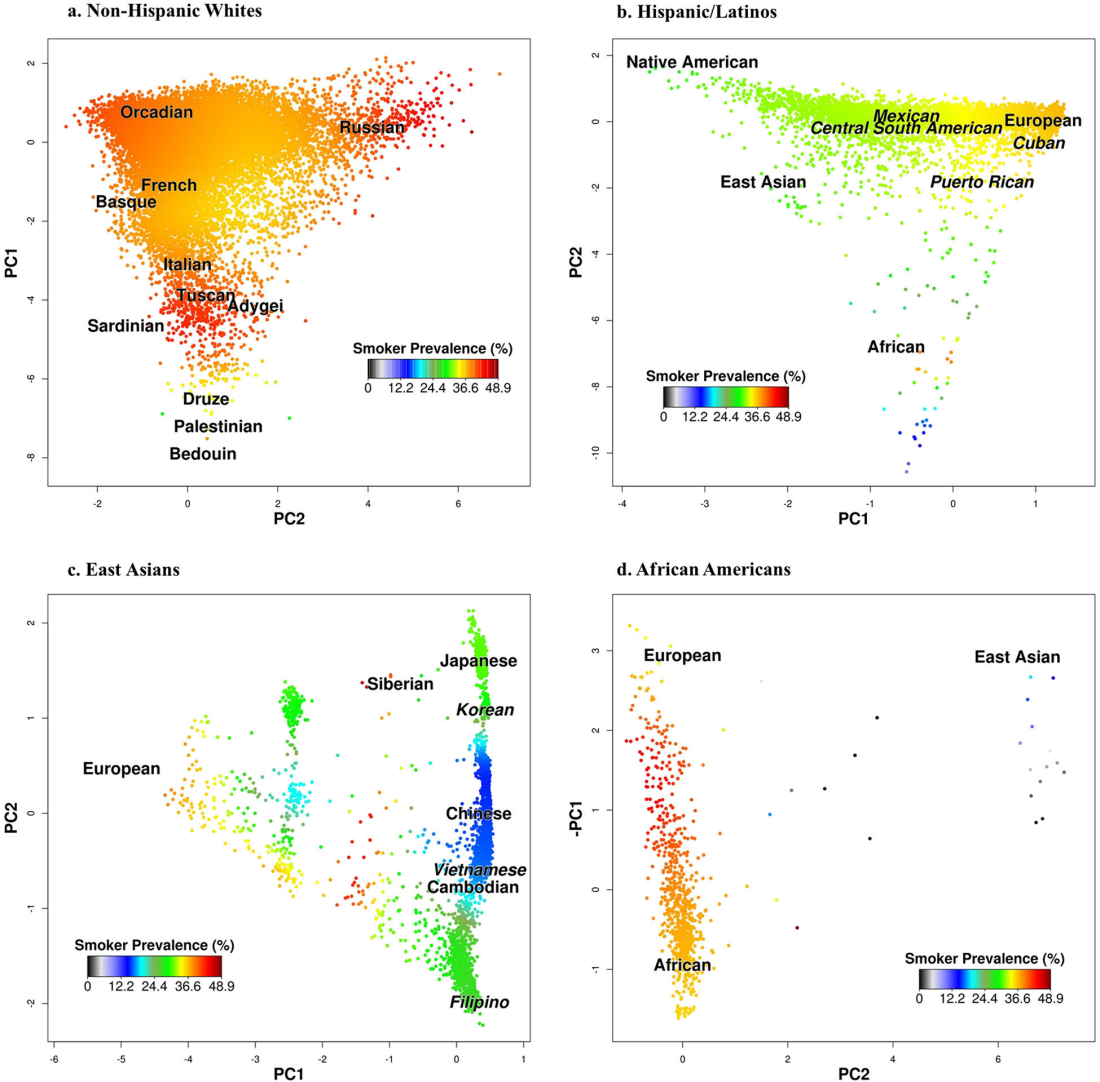
Smoking Initiation Prevalence vs. Genetic Ancestry. Plots of smoking initiation prevalence vs. genetic ancestry in GERA: (**a**) non-Hispanic whites, (**b**) Hispanic/Latinos, (**c**) East Asians, and (**d**) African Americans. Human Genome Diversity Panel populations are plotted at their relative positions.

**Fig. 2:**
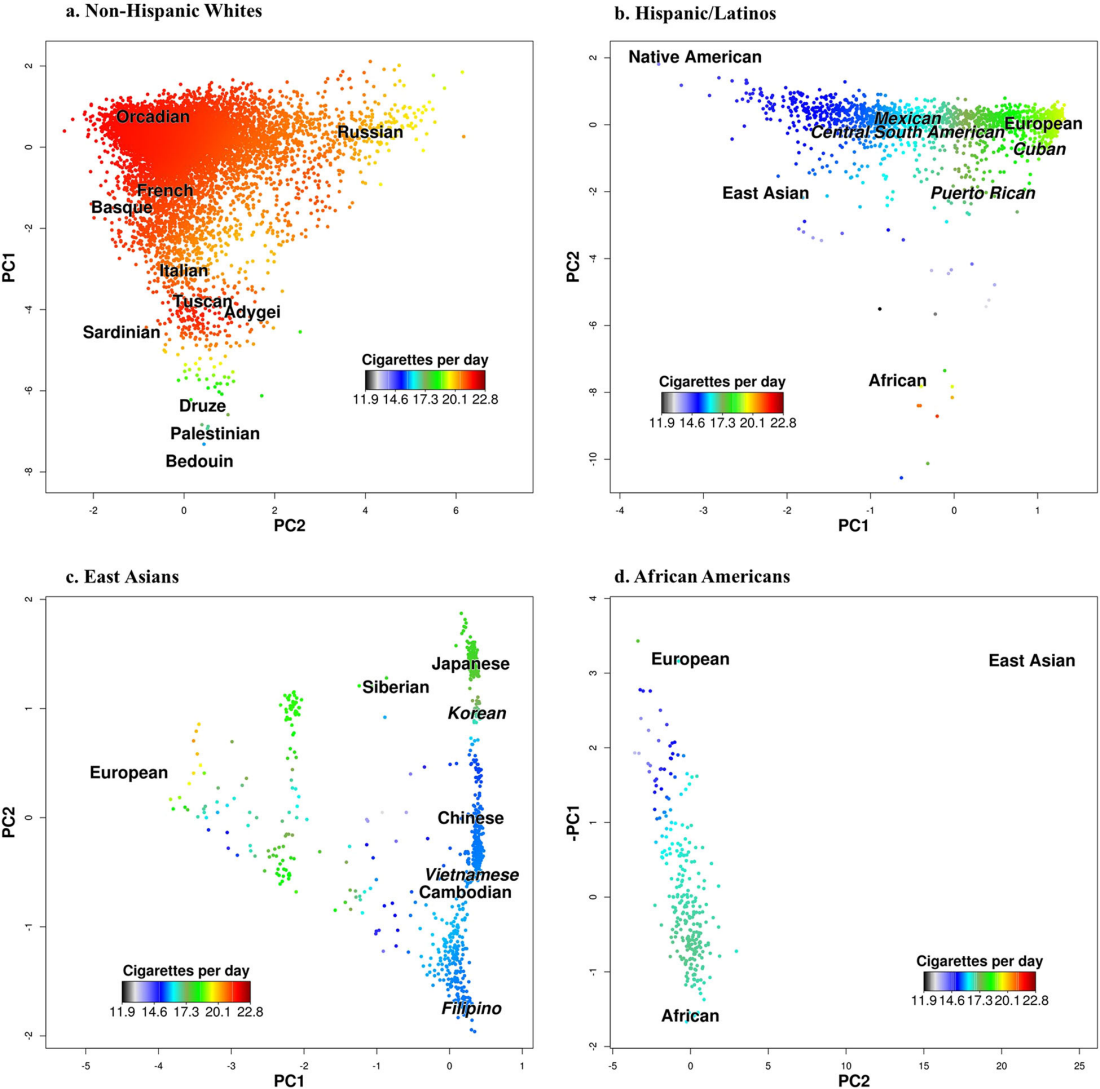
Number of Cigarettes Smoked Per Day (CPD) Distribution vs. Genetic Ancestry. Plots of the number of CPD distribution vs. genetic ancestry in GERA: (**a**) non-Hispanic whites, (**b**) Hispanic/Latinos, (**c**) East Asians, and (**d**) African Americans. Human Genome Diversity Panel populations are plotted at their relative positions.

We first investigated genome-wide genetic ancestry using principal components (PCs) that were assessed within each ethnic group separately^16^. Genetic ancestry associations with smoking initiation and CPD were then assessed and visual representations are provided in Figs. 1, 2. Within non-Hispanic whites, the first two PCs represented geographically interpretable genetic ancestry, with PC1 characterizing a northwestern vs. southeastern European cline and PC2 a northeastern vs. southwestern European cline. The first two PCs were both associated with CPD (Model 1: *β* = 27.95, *P*_PC1_ = 0.017; *β* = −50.32, *P*_PC2_ = 9.06 × 10^−5^) (Table 2), with the lowest prevalence observed for individuals of southeastern European ancestry (Fig. 2a). In contrast, neither PC1 nor PC2 was associated with smoking initiation within non-Hispanic whites.

**Table 2:** Associations between genetic ancestry and smoking-related phenotypes in GERA non-Hispanic whites and Hispanic/Latinos.

	GERA non-Hispanic whites				GERA Hispanic/Latinos			
	Smoking initiation		CPD (within all smokers)		Smoking initiation		CPD (within all smokers)	
	*β* (SE)	*P*-value	*β* (SE)	*P*-value	*β* (SE)	*P*-value	*β* (SE)	*P*-value
*Model 1: Ancestry (age, sex, and PCs as covariates)*								
PC1	0.80 (1.63)	0.63	27.95 (11.68)	0.017	17.67 (4.02)	1.12 × 10^−5^	271.29 (25.66)	3.34 × 10^−25^
PC2	−3.35 (1.84)	0.07	−50.32 (12.85)	9.06 × 10^−5^	4.60 (3.22)	0.15	17.30 (22.10)	0.43
*Model 2: Model 1 and genetic risk score*								
PC1	0.75 (1.65)	0.65	34.07 (11.61)	3.34 × 10^−3^	22.80 (4.15)	4.07 × 10^−8^	263.32 (25.96)	2.18 × 10^−23^
PC2	−3.89 (1.85)	0.04	−50.90 (12.76)	6.69 × 10^−5^	4.83 (3.22)	0.13	19.21 (22.10)	0.38
GRS	1.02 (0.05)	2.81 × 10^−94^	6.72 (0.50)	2.25 × 10^−40^	0.79 (0.15)	8.28 × 10^−8^	2.72 (1.40)	0.052
*Model 3: Model 2 and socioeconomic factors*								
PC1	2.24 (1.68)	0.18	39.51 (11.51)	6.02 × 10^−4^	27.85 (4.35)	1.58 × 10^−10^	248.22 (26.95)	1.14 × 10^−19^
PC2	−2.70 (1.88)	0.15	−46.06 (12.65)	2.74 × 10^−4^	4.76 (3.30)	0.15	18.99 (22.14)	0.39
GRS	0.96 (0.05)	5.44 × 10^−81^	6.73 (0.50)	3.62 × 10^−41^	0.76 (0.15)	2.87 × 10^−7^	2.89 (1.40)	0.040
Education								
Less than high school	0.36 (0.33)	0.27	7.16 (2.09)	6.05 × 10^−4^	0.17 (0.23)	0.44	1.04 (1.46)	0.48
High school	0.80 (0.048)	7.39 × 10^−63^	2.76 (0.29)	2.12 × 10^−21^	0.61 (0.10)	9.17 × 10^−10^	−0.06 (0.61)	0.92
Some college	0.56 (0.028)	4.09 × 10^−85^	2.02 (0.19)	8.74 × 10^−27^	0.43 (0.08)	5.59 × 10^−8^	0.09 (0.50)	0.86
Employment								
Disabled	0.33 (0.072)	3.31 × 10^−6^	2.48 (0.42)	4.54 × 10^−9^	0.04 (0.19)	0.83	2.28 (1.14)	0.047
Unemployed	0.17 (0.083)	0.037	−0.11 (0.53)	0.84	−0.09 (0.24)	0.70	1.80 (1.44)	0.21
Part-time	−0.16 (0.032)	4.81 × 10^−7^	−0.20 (0.22)	0.38	−0.12 (0.10)	0.24	0.94 (0.66)	0.15
Income								
<$20,000	−0.01 (0.089)	0.87	−0.45 (0.57)	0.43	0.11 (0.08)	0.19	−0.80 (0.52)	0.13
$20,000–$59,999	0.16 (0.03)	1.94 × 10^−6^	−0.32 (0.22)	0.15	0.24 (0.18)	0.19	−1.62 (1.11)	0.15
Marital status								
Never married	0.05 (0.043)	0.22	0.87 (0.30)	4.12 × 10^−3^	−0.03 (0.11)	0.76	1.40 (0.71)	0.0496
Separated/divorced	0.25 (0.035)	1.26 × 10^−12^	0.69 (0.23)	2.31 × 10^−3^	0.13 (0.10)	0.18	1.54 (0.62)	0.012

Note: In model 3, sex (female), education (college or more), income ($60,000 or more), marital status (married or living as married), employment (full-time employed) served as the reference group. Each model was adjusted for age, sex, and additional PCs. We also included the percentage of Ashkenazi (ASHK) ancestry as a covariate for the non-Hispanic white analyses.

*CPD* number of cigarettes smoked per day, *PC* principal component, *β* beta, *SE* standard error, *GRS* genetic risk score (based on 365 SNPs previously reported to be associated with smoking initiation, or 53 SNPs previously reported to be associated with CPD).

Within Hispanic/Latinos, the first two PCs were also geographically interpretable, with PC1 representing greater European versus Native American ancestry and PC2 representing greater African versus European ancestry. In Hispanic/Latinos, we observed higher smoking initiation prevalence and higher CPD correlating with greater European (versus Native American) ancestry (Model 1: *β* = 17.67, *P*_PC1_ = 1.12 × 10^−5^ for smoking initiation; and *β* = 271.29, *P*_PC1_ = 3.34 × 10^−25^ for CPD) (Table 2**;** Figs. 1b and 2b).

In East Asians, PC1, which represents European admixture, was strongly associated with smoking initiation (Model 1: *β* = −23.15, *P*_PC1_ = 9.95 × 10^−12^) and nominally with CPD (Model 1: *β* = −48.22, *P*_PC1_ = 0.03). For PC2, which differentiates geographical clines across East Asia, we observed a non-linear association between smoking initiation and PC2 (Model 1: *β* = 10.12, *P*_PC2_ = 0.011 for smoking initiation). This non-linear association represents a U-shaped association of ancestry from north to south (or south to north) (Table 3**;** Fig. 1c). Recently, we reported a similar pattern of ancestry association for body mass index in East Asians^27^. Significant associations were also detected between PC2 and CPD (Model 1: *β* = 66.74, *P*_PC2_ = 3.92 × 10^−3^) (Fig. 2c).

**Table 3:** Associations between genetic ancestry and smoking-related phenotypes in GERA East Asians and African Americans.

Ethnic group	GERA East Asians				GERA African Americans			
Smoking phenotype	Smoking initiation		CPD (within all smokers)		Smoking initiation		CPD (within all smokers)	
	*β* (SE)	*P*-value	*β* (SE)	*P*-value	*β* (SE)	*P*-value	*β* (SE)	*P*-value
*Model 1: Ancestry (age, sex, and PCs as covariates)*								
PC1	−23.15 (3.40)	9.95 × 10^−12^	−48.22 (21.75)	0.03	−0.52 (3.01)	0.86	−4.81 (19.47)	0.81
PC2	10.12 (3.97)	0.011	66.74 (23.08)	3.92 × 10^−3^	3.12 (3.20)	0.33	19.46 (19.32)	0.31
PC2^2^	−729.48 (835.53)	0.38	–	–	–	–	–	–
*Model 2: Model 1 and genetic risk score*								
PC1	−24.06 (3.42)	2.05 × 10^−12^	−31.97 (22.43)	0.15	−1.59 (3.05)	0.60	−4.55 (19.31)	0.81
PC2	9.10 (3.99)	0.022	66.22 (22.99)	4.07 × 10^−3^	3.43 (3.21)	0.29	19.26 (19.16)	0.32
PC2^2^	−717.78 (836.27)	0.39	–	–	–	–	–	–
GRS	0.57 (0.20)	3.60 × 10^−3^	7.29 (2.60)	5.14 × 10^−3^	0.59 (0.28)	0.03	8.81 (2.85)	2.09 × 10^−3^
*Model 3: Model 2 and socioeconomic factors*								
PC1	−19.97 (3.51)	1.26 × 10^−8^	−23.74 (22.69)	0.30	−3.83 (3.12)	0.22	−5.57 (20.29)	0.78
PC2	9.51 (4.09)	0.020	60.76 (23.30)	9.27 × 10^−3^	3.56 (3.26)	0.27	18.84 (19.48)	0.33
PC2^2^	−502.64 (852.21)	0.56	–	–	–	–	–	–
GRS	0.57 (0.20)	4.21 × 10^−3^	7.92 (2.60)	2.36 × 10^−3^	0.50 (0.28)	0.072	8.71 (2.90)	2.80 × 10^−3^
Education								
Less than high school	0.20 (0.44)	0.65	0.67 (3.20)	0.83	0.30 (0.74)	0.68	0.53 (4.08)	0.90
High school	0.57 (0.16)	3.98 × 10^−4^	1.51 (1.06)	0.15	0.68 (0.22)	1.89 × 10^−3^	−0.13 (1.23)	0.91
Some college	0.95 (0.10)	1.00 × 10^−19^	1.04 (0.65)	0.11	0.41 (0.13)	1.15 × 10^−3^	0.98 (0.77)	0.21
Employment								
Disabled	−0.18 (0.40)	0.64	−2.77 (2.56)	0.28	0.25 (0.31)	0.41	0.03 (1.66)	0.98
Unemployed	0.45 (0.24)	0.058	0.52 (1.57)	0.74	0.21 (0.38)	0.58	1.02 (2.32)	0.66
Part-time	0.13 (0.13)	0.32	−1.59 (0.91)	0.08	−0.48 (0.21)	0.021	−0.15 (1.31)	0.91
Income								
<$20 000	0.25 (0.26)	0.34	−3.48 (1.74)	0.045	0.03 (0.37)	0.94	−1.21 (2.08)	0.56
$20,000–$59, 999	0.10 (0.11)	0.37	−0.81 (0.71)	0.26	0.19 (0.14)	0.16	−0.22 (0.84)	0.80
Marital status								
Never married	−0.04 (0.14)	0.76	−0.38 (1.0)	0.71	0.10 (0.18)	0.59	−0.83 (1.15)	0.47
Separated/divorced	0.56 (0.13)	2.91 × 10^−5^	2.09 (0.86)	0.015	0.12 (0.14)	0.40	−0.02 (0.88)	0.98

Note: In East Asians, because of the non-linear effect, we included a quadratic term (PC22) in the models for smoking initiation.

*CPD* number of cigarettes smoked per day, *PC* principal component, *β* beta, *SE* standard error, *GRS* genetic risk score (based on 365 SNPs previously reported to be associated with smoking initiation, or 53 SNPs previously reported to be associated with CPD). In model 3, sex (female), education (college or more), income ($60,000 or more), marital status (married or living as married), employment (full-time employed) served as the reference group. Each model was adjusted for age, sex, and additional PCs.

In African Americans, neither PC1 (representing African vs. European ancestry) nor PC2 (representing East Asian ancestry) were associated with smoking initiation or CPD (Table 3**;** Figs. 1d and 2d).

### Genetic ancestry and known smoking-associated loci

To determine whether the genetic ancestry associations with smoking-related traits were due to known smoking-associated loci, we repeated the ancestry analyses, including one of the two following GRS: the first GRS was based on 365 smoking initiation associated-SNPs, and the second GRS was based on 53 SNPs previously reported to be associated with CPD^15^. While the GRS for smoking initiation was significantly associated with smoking initiation in all four ethnic groups, the GRS for CPD was a predictor for CPD in all ethnic groups, except Hispanic/Latinos (Table 2).

In non-Hispanic whites, the genetic ancestry associations between PC1 or PC2 and CPD were not attenuated after including the GRS for CPD (Model 2: *β* = 34.07, *P*_PC1_ = 3.34 × 10^−3^; *β* = −50.90, *P*_PC2_ = 6.69 × 10^−5^) (Table 2). In Hispanic/Latinos, while the genetic ancestry association between PC1 and smoking initiation was not attenuated when including a GRS, the genetic association between PC1 and CPD was slightly attenuated (Model 2: *β* = 22.80, *P*_PC1_ = 4.07 × 10^−8^ for smoking initiation; *β* = 263.32, *P*_PC1_ = 2.18 × 10^−23^ for CPD) (Table 2). In East Asians, while the genetic ancestry association between PC1 and smoking initiation was not attenuated when including a GRS, the genetic ancestry association between PC2 and smoking initiation was slightly attenuated (Model 2: *β* = −24.06, *P*_PC1_ = 2.05 × 10^−12^; *β* = 9.10, *P*_PC2_ = 0.022 for smoking initiation) (Table 3). Further, in East Asians, while the genetic ancestry association between PC1 and CPD was no longer significant when including a GRS, the genetic ancestry association between PC2 and CPD was slightly attenuated (Model 2: *β* = −31.97, *P* = 0.15 for PC1 and *β* = 66.22, *P* = 4.07 × 10^−3^ for PC2) (Table 3).

### Genetic ancestry associations and socioeconomic factors

To determine whether education, socioeconomic factors, and marital status explain the remaining genetic ancestry associations (after considering genetically determined smoking-related traits), we repeated the ancestry analyses, including education, employment, income level, and marital status. In non-Hispanic whites, only the genetic ancestry association between PC2 and CPD was attenuated after considering education, socioeconomic factors, and marital status (Model 3: *β* = −46.06, *P*_PC2_ = 2.74 × 10^−4^) (Table 2). In Hispanic/Latinos, while the genetic ancestry association between PC1 and smoking initiation was not attenuated when considering education, socioeconomic factors, and marital status, the genetic association between PC1 and CPD was attenuated further but not eliminated (Model 3: *β* = 27.85, *P*_PC1_ = 1.58 × 10^−10^ for smoking initiation; *β* = 248.22, *P*_PC1_ = 1.14 × 10^−19^ for CPD) (Table 2). In East Asians, the genetic ancestry association between PC1 and smoking initiation was attenuated when considering education, socioeconomic factors, and marital status, and the genetic ancestry association between PC2 and CPD was attenuated further but not eliminated (Model 3: *β* = −19.97, *P*_PC1_ = 1.26 × 10^−8^ for smoking initiation and *β* = 60.76, *P*_PC2_ = 9.27 × 10^−3^ for CPD) (Table 3).

## Discussion

In this study, we observed substantial differences in cigarette smoking behaviors across race/ethnicity groups, and we found that smoking initiation and/or CPD were associated with genetic ancestry within non-Hispanic whites, Hispanic/Latinos, and East Asians. Specifically, a higher smoking initiation prevalence and higher number of CPD were associated with greater European (versus Native American) ancestry among Hispanic/Latinos and were associated with greater European (versus Asian) ancestry among East Asians. Furthermore, individuals of northwestern European ancestry had a higher number of CPD compared to individuals of southeastern European ancestry among non-Hispanic whites. No significant associations between genetic ancestry and cigarette smoking behaviors were detected in African Americans, which was the smallest sample size of the groups. After considering genetic variants known to contribute to cigarette smoking behaviors and accounting for education, socioeconomic factors such as employment/work status and household income, and marital status, these genetic ancestry associations remained, but were attenuated. Study findings suggest that genetically determined smoking traits and socioeconomic factors can explain some of the ancestry effects in Hispanic/Latinos, East Asians, and non-Hispanic whites, and that additional factors correlated with genetic ancestry remain to be discovered.

Our results are consistent with previous studies showing disparities in adult cigarette smoking prevalence among specific sub-populations, including individuals from certain ethnic groups, variation by education level, and socioeconomic groups. Indeed, we found that East Asian and Hispanic/Latino individuals had the lowest prevalence of smoking initiation compared to non-Hispanic white and African American individuals, consistent with the previous studies^7,28^. Similarly, in our study, the prevalence of these ‘ever’ smokers was much lower for college-educated individuals compared to those with high school education, and for individuals who earned >$60,000 compared to those with lower income, consistent with previous studies^7,28–30^. Furthermore, in our study, married individuals had the highest prevalence of smoking cessation compared to those who were single or divorced, consistent with previous findings^31^.

We recognize several potential limitations of our study. First, the cigarette smoking-related traits were based on self-reported information, and no information regarding other forms of tobacco use, such as pipes, cigars, or e-cigarettes, were collected on our survey. Further, GERA cohort members are older on average compared to the general population. As older adults may consume tobacco in a different form than younger adults who may prefer e-cigarettes^32,33^, this may limit the generalizability of the findings to the groups represented in this study. Second, no information regarding the previous U.S. addresses of the participants included in the current study was collected. All the GERA members were living in the Northern California region at the time of survey completion, however, as smoking prevalence has been shown to vary considerably across states^7,34^, considering the previous U.S. addresses of the participants could identify an additional potential source of variation in smoking behavior. Third, because of the limited number of ‘current’ smokers in our sample (*N* = 2271), we did not consider the smoking cessation phenotype (i.e., ‘current’ vs. ‘former’ smokers) for the subsequent genetic ancestry association analyses. Lastly, for the calculation of GRS for smoking-related traits, we used a ‘classic’ GRS method^26^ that restricts to only genetic variants reaching genome-wide significance in the original GWAS^15^. This ‘classic’ approach has been commonly applied^35–39^ and has key advantages^26^, including that it is relatively fast to apply and is more interpretable compared to more sophisticated methods, such as Bayesian regression models that perform shrinkage^39–41^. Further, this ‘classic’ approach has been shown to have relatively similar performance compared to alternative methods^39–41^. Future studies applying those alternative methods to derive GRS for smoking-related traits may provide a further refinement to the effects that we observed in the current study. Despite these limitations, our study is based on a unique and very large cohort of individuals, who were all members of the KPNC health plan, a single integrated healthcare delivery system. Participants were recruited in a similar manner and were assessed for their cigarette smoking behaviors using a single questionnaire providing greater consistency, in contrast to consortia which often include different questions across studies.

In conclusion, this study is the first investigation of genetic ancestry and cigarette smoking-related trait associations. We observed significant associations between genetic ancestry and smoking-related traits within each race/ethnicity, except for African Americans. Known smoking-associated genetic variants identified in populations of European ancestry^15^ explained only a small proportion of these associations, and the observed ancestry effects may be due to population-specific genetic variants. Future studies including additional genetic variants associated with smoking behavior-related traits in non-European populations, such as those recently identified in a Japanese population^42^ but not validated yet, may better explain these genetic ancestry associations.

## Supplementary information

Supplementary Information

Supplementary Data

## Data Availability

Genotype data of GERA participants are available from the database of Genotypes and Phenotypes (dbGaP) under accession phs000674.v2.p2. This includes individuals who consented to having their data shared with dbGaP. The complete GERA data are available upon application to the KP Research Bank (https://researchbank.kaiserpermanente.org/).
