# Supplementary material for: Cigarette smoking behaviors and the importance of ethnicity and genetic ancestry
Source: Transl Psychiatry. 2021 Feb 11;11:120. doi: 10.1038/s41398-021-01244-7 (PMC7907280; doi:10.1038/s41398-021-01244-7)
Supplement: Supplementary file 1 — Supplementary Information [file 41398_2021_1244_MOESM1_ESM.docx]

**Supplementary Table 1.** Characteristics of the GERA participants included in the current study by smoking status

|  | **Ever smoker** | | | **Within smoker** | | |
| --- | --- | --- | --- | --- | --- | --- |
|  | **Smoker** | **Non-smoker** | ***P*-value** | **Current smoker** | **Former smoker** | ***P*-value** |
| **All, n (%)** | 15,862 (36.5%) | 27,623 |  | 2,271 (14.3%) | 13,591 |  |
| **Gender, n (%)** |  |  |  |  |  |  |
| Female | 8,241 (32.8%) | 16,899 | 2.52 x 10^-78^ | 1,160 (14.1%) | 7,081 | 0.56145 |
| Male | 7,621 (41.5%) | 10,724 |  | 1,111 (14.6%) | 6,510 |  |
| **Age at specimen, mean ± sd** | 58.3 ± 10.1 | 52.8 ± 11.5 | <2.23x10^-308^ | 54.9 ± 11.0 | 58.9 ± 9.8 | 1.48x10^-55^ |
| **Race/Ethnicity** |  |  |  |  |  |  |
| Non-Hispanic White | 12,898 (38.5%) | 20,640 | 2.27 x 10^-86^ | 1,717 (13.3%) | 11,181 | 1.48 x 10^-16^ |
| Hispanic/Latino | 1,489 (33.9%) | 2,903 |  | 280 (18.8%) | 1,209 |  |
| African American | 554 (36.9%) | 949 |  | 132 (23.8%) | 422 |  |
| East Asian | 921 (22.7%) | 3,131 |  | 142 (15.4%) | 779 |  |
| **Education** |  |  |  |  |  |  |
| Less than high school | 70 (36.8%) | 120 | 2.39 x 10^-215^ | 19 (27.1%) | 51 | 3.11 x 10^-48^ |
| High school | 1,653 (51.3%) | 1,569 |  | 370 (22.4%) | 1,283 |  |
| Some college | 4,548 (46.1%) | 5,310 |  | 814 (17.9%) | 3,734 |  |
| College degree or more | 9,591 (31.7%) | 20,624 |  | 1,068 (11.1%) | 8,523 |  |
| **Employment** |  |  |  |  |  |  |
| Full-time employed | 11,986 (36.1%) | 21,245 | 1.44 x 10^-39^ | 1,793 (15.0%) | 10,193 | 5.63 x 10^-17^ |
| Part-time employed | 2,819 (34.8%) | 5,280 |  | 271 (9.6%) | 2,548 |  |
| Disabled | 660 (53.5%) | 574 |  | 129 (19.5%) | 531 |  |
| Unemployed | 397 (43.1%) | 524 |  | 78 (19.6%) | 319 |  |
| **Marital Status** |  |  |  |  |  |  |
| Never Married | 1,514 (28.9%) | 3,727 | 3.60 x 10^-82^ | 338 (22.3%) | 1,176 | 2.50 x 10^-33^ |
| Married or living as married | 11,389 (35.8%) | 20,385 |  | 1,400 (12.3%) | 9,989 |  |
| Separated / Divorced | 2,959 (45.7%) | 3,511 |  | 533 (18.0%) | 2,426 |  |
| **Income Level** |  |  |  |  |  |  |
| <$20,000 | 458 (43.2%) | 603 | 3.08 x 10^-57^ | 119 (26.0%) | 339 | 3.99 x 10^-39^ |
| $20,000-$59,999 | 3,674 (43.6%) | 4,759 |  | 720 (19.6%) | 2,954 |  |
| $60,000+ | 11,730 (34.5%) | 22,261 |  | 1,432 (12.2%) | 10,298 |  |
| **Cigarettes per day, mean ± sd** | 20.4 ± 9.4 |  | - | 18.3 ± 8.3 | 20.7 ± 9.5 | 2.80 x 10^-34^ |

**Supplementary Table 2.** Characteristics of the GERA participants included in the current study by race/ethnicity

|  | **NHW**  **(n = 33,538)** | | | **H/L**  **(n = 4,392)** | | | **EAS**  **(n = 4,052)** | | | **AA**  **(n = 1,503)** | | |
| --- | --- | --- | --- | --- | --- | --- | --- | --- | --- | --- | --- | --- |
|  | **Smoker**  **(n=12,898)** | **CPD mean ± sd** | **Non-smoker**  **(n=20,640)** | **Smoker**  **(n=1,489)** | **CPD mean ± sd** | **Non-smoker**  **(n=2,903)** | **Smoker**  **(n=921)** | **CPD mean ± sd** | **Non-smoker**  **(n=3,131)** | **Smoker**  **(n=554)** | **CPD mean ± sd** | **Non-smoker**  **(n=949)** |
| **Education** |  |  |  |  |  |  |  |  |  |  |  |  |
| Less than high school | 20 (51.3) | 28.4 ± 10.7 | 19 | 38 (36.2) | 16.3 ± 9.7 | 67 | 8 (21.1) | 15.7 ± 11.3 | 30 | 4 (50.0) | 17.5 ± 15.0 | 4 |
| High school | 1,211 (55.6) | 23.0 ± 9.3 | 966 | 301 (44.3) | 16.4 ± 8.3 | 378 | 82 (48.2) | 17.4 ± 8.8 | 170 | 59 (51.8) | 16.5 ± 8.0 | 55 |
| Some college | 3,625 (48.1) | 22.4 ± 9.6 | 3,909 | 478 (38.2) | 17.1 ± 8.5 | 774 | 249 (40.9) | 17.1 ± 8.7 | 360 | 196 (42.3) | 17.8 ± 8.7 | 267 |
| College degree or more | 8,042 (33.8) | 20.3 ± 9.2 | 15,746 | 672 (28.5) | 17.2 ± 8.7 | 1,684 | 582 (18.5) | 16.0 ± 8.2 | 2,571 | 295 (32.1) | 16.7 ± 7.5 | 623 |
| **Employment** |  |  |  |  |  |  |  |  |  |  |  |  |
| Full-time employed | 9,601 (38.0) | 21.1 ± 9.4 | 15,638 | 1,164 (33.8) | 16.5 ± 8.3 | 2,275 | 764 (23.1) | 16.7 ± 8.5 | 2,548 | 457 (36.8) | 17.1 ± 8.1 | 784 |
| Part-time employed | 2,429 (36.6) | 20.8 ± 9.3 | 4,202 | 227 (31.7) | 18.6 ± 9.3 | 489 | 112 (19.0) | 15.0 ± 7.2 | 476 | 51 (31.1) | 16.9 ± 7.4 | 113 |
| Disabled | 554 (56.1) | 24.2 ± 9.8 | 433 | 63 (44.4) | 19.8 ± 9.0 | 79 | 11 (26.8) | 14.5 ± 6.9 | 30 | 32 (50.0) | 17.1 ± 8.2 | 32 |
| Unemployed | 314 (46.1) | 21.2 ± 9.4 | 367 | 35 (36.8) | 18.2 ± 9.0 | 60 | 34 (30.6) | 15.8 ± 9.4 | 77 | 14 (41.2) | 17.7 ± 9.3 | 20 |
| **Marital Status** |  |  |  |  |  |  |  |  |  |  |  |  |
| Never Married | 1,147 (31.7) | 20.8 ± 9.6 | 2,470 | 188 (26.6) | 17.5 ± 9.0 | 518 | 101 (15.8) | 14.4 ± 7.6 | 537 | 78 (27.9) | 15.6 ± 7.4 | 202 |
| Married or living as married | 9,320 (37.5) | 21.1 ± 9.4 | 15,504 | 1,051 (34.5) | 16.7 ± 8.3 | 1,998 | 696 (22.8) | 16.4 ± 8.5 | 2,351 | 322 (37.7) | 17.4 ± 8.1 | 532 |
| Separated / Divorced | 2,431 (47.7) | 21.6 ± 9.4 | 2,666 | 250 (39.2) | 17.9 ± 9.2 | 387 | 124 (33.8) | 18.1 ± 8.5 | 243 | 154 (41.7) | 17.0 ± 8.2 | 215 |
| **Income Level** |  |  |  |  |  |  |  |  |  |  |  |  |
| <$20,000 | 327 (45.9) | 22.6 ± 9.9 | 386 | 77 (41.0) | 17.4 ± 8.0 | 111 | 33 (30.8) | 13.8 ± 6.2 | 74 | 21 (39.6) | 15.2 ± 6.8 | 32 |
| $20,000-$59,999 | 2,809 (46.9) | 21.7 ± 9.5 | 3,184 | 451 (37.1) | 16.5 ± 8.5 | 766 | 227 (29.2) | 16.2 ± 8.0 | 550 | 187 (41.9) | 17.1 ± 8.1 | 259 |
| $60,000+ | 9,762 (36.4) | 21.0 ± 9.4 | 17,070 | 961 (32.2) | 17.2 ± 8.6 | 2,026 | 661 (20.9) | 16.6 ± 8.6 | 2,507 | 346 (34.5) | 17.2 ± 8.1 | 658 |
